# Histone Methyltransferases as Therapeutic Targets for Kidney Diseases

**DOI:** 10.3389/fphar.2019.01393

**Published:** 2019-12-06

**Authors:** Chao Yu, Shougang Zhuang

**Affiliations:** ^1^Department of Nephrology, Shanghai East Hospital, Tongji University School of Medicine, Shanghai, China; ^2^Department of Medicine, Rhode Island Hospital and Alpert Medical School, Brown University, Providence, RI, United States

**Keywords:** chronic kidney diseases, epigenetic regulations, histone modification, histone methyltransferases, expression

## Abstract

Emerging evidence has demonstrated that epigenetic regulation plays a vital role in gene expression under normal and pathological conditions. Alterations in the expression and activation of histone methyltransferases (HMTs) have been reported in preclinical models of multiple kidney diseases, including acute kidney injury, chronic kidney disease, diabetic nephropathy, polycystic kidney disease, and renal cell carcinoma. Pharmacological inhibition of these enzymes has shown promise in preclinical models of those renal diseases. In this review, we summarize recent knowledge regarding expression and activation of various HMTs and their functional roles in some kidney diseases. The preclinical activity of currently available HMT inhibitors and the mechanisms of their actions are highlighted.

## Introduction

Epigenetics is a heritable change in gene expression, including covalent modifications of DNA and related histone proteins in the absence of an altered change in DNA sequence. In general, epigenetic regulation involving histone modification, DNA methylation, and non-coding RNAs can alter the phenotype but not the genotype in gene expression (Peschansky and Wahlestedt, 2014; Reddy and Natarajan, 2015). Histone modification involves distinct types of post-translational modifications (PTMs) of core histone proteins, including acetylation, methylation, phosphorylation, and ubiquitination (Susztak, 2014). These modifications occur primarily on the amino terminal ends of the histones within the nucleosome core. They are thought to change the structure and transcriptional process of chromatin, thereby influencing the downstream process of gene expression positively or negatively, alone or in combination with other modifications of gene expression (Chen et al., 2014b).

Histone methylation commonly occurs in specific lysine and arginine residues at the amino terminal ends of histones core (Greer and Shi, 2012). Each lysine has three possible methylation states: mono-methylated, di-methylated or tri-methylated with methyl groups covalently bonded to the lysine ε-amine group. In contrast, arginine post-methylation can exist in mono-methylated, di-methylated symmetrical and asymmetrical forms with methylation of the molecule’s guanidyl group (Bedford and Richard, 2005). Differences in residue methylation and modification states correlate with whether histone methylation activates or represses transcription (Li et al., 2007). For example, lysine methylation at H3 lysine 4 (H3K4), H3K36, and H3K79 is associated with transcriptional activation. Conversely, methylation at H3K9, H3K27, and H4K20 is related to gene repression (Sims et al., 2003). The underlying mechanism by which the active and repressive actions can operate in such a coordinated manner remains incompletely understood.

Histone methyltransferases (HMTs) are a class of enzymes that mediate the methylation of lysine or arginine residues of histones. So far, more than 50 lysine human methyltransferases (KMTs) have been reported. These enzymes have high selectivity for the histone lysine residue they target and are classified into two types: lysine methyltransferases (KMTs) and arginine methyltransferases (PRMTs). Based on catalytic domain sequence, KMTs are further divided into two families: SET domain-containing KMTs, which include Su(var)3-9, Enhancer of Zeste (EZH), Trithorax, and non-SET domain-containing KMTs, such as the DOT1-like proteins (Feng et al., 2002; Ng et al., 2002; Herz et al., 2013). The structure of SET methyltransferase contains a SET domain, a pre-SET, and a post-SET domain. SET methyltransferases are further sub-divided into different families. The SET1 family bears the SET domain usually followed by a post-SET domain. Two well-known methyltransferases, EZH1, and EZH2, belong to this family although they do not have the post-SET domain. The SET domain in the SET2 family is always flanked by a post-SET and an AWS domain, where the nuclear receptor binds to the SET domain, which contains proteins such as NSD1-3, the SETD2 and the SMYD family proteins. Members of the SUV39 family all demonstrate a pre-SET domain that includes SUV39H1, SUV39H2, G9a, GLP, ESET, and CLLL8 (Rea et al., 2000). Still other SET domain-containing methyltransferases have not been categorized into specific sub-groups, like SET7/9, SET8, SUV4-20H1, and SUV4-20H2 (Dillon et al., 2005). The human DOT1-like (DOT1L) protein is a non-SET domain containing methyltransferase that methylates a lysine residue in the globular core of the histone (Wood and Shilatifard, 2004). Among the PRMTs, there are three types of methylation patterns based on different arginine binding pockets. The first type of PRMTs, including PRMT1, PRMT3, and cofactor associated arginine methyltransferase (CARM1), can produce monomethyl arginine and asymmetric dimethylarginine (Chen et al., 1999; McBride et al., 2000). The second type of PRMTs, including PRMT5, can produce monomethyl or symmetric dimethylarginine (Branscombe et al., 2001). The third type of PRMTs, including PRMT7, only produces monomethylated arginine (Blanc and Richard, 2017) (**Table 1**).

**Table 1:** Classification of histone methyltransferases.

Methyltransferase		Type		Site	Function
KMTs		SET1 family	EZH1	H3K27	Transcriptional repression
		SET1 family	H3K27	H2K27	Transcriptional repression
		SET2 family	NSD1-3	H3K36	Transcriptional activation
		SET2 family	SETD2	H3K36	Transcriptional activation
		SET2 family	SMYD2	H3K36/p53	Transcriptional activation
		SUV39 family	SUV39H1	H3K9	Heterochromatin formation/silencing
		SUV39 family	SUV39H2	H3K9	Heterochromatin formation/silencing
		SUV39 family	G9a	H3K9	Heterochromatin formation/silencing
		SUV39 family	GLP	H3K9	Heterochromatin formation/silencing
		SUV39 family	ESET//SETDB1	H3K9	Transcriptional repression
		SUV39 family	CLLL8/SETDB2		
		RIZ family	RIZ1	H3K9	Transcriptional repression
		RIZ family	BLIMP1/PRDM1		
		RIZ family	PFM1/CRS2		
		Non-group family	SET7/9	H3K4/p53/TAF10	Transcriptional repression
		Non-group family	SET8	H4K20	Transcriptional repression
		Non-group family	SUV4-20H1	H4K20	DNA damage response
		Non-group family	SUV4-20H2	H4K20	
		non-SET family	DOT1L	H3K79	Transcriptional activation
PRMTs		I	PRMT1	H4R3	Transcriptional activation
		I	PRMT3	RPS2	Transcriptional activation
		I	PRMT4 /CARM1	H3R2, H3R17, H3R26	Transcriptional activation
		II	PRMT5	H3R8, H4R3	Transcriptional repression
		III	PRMT7	H4R3	Imprinting in male germ cell

Histone methylation plays important roles in many biological processes, including cell cycle regulation, stress response, development, and differentiation. Initial studies indicated that H3 methylation on lysines 4, 27, and 79 plays an essential part in regulation of gene expression in the developing kidney (McLaughlin et al., 2014). Recent studies have demonstrated that abnormal expression or activity of histones methyltransferases are associated with development and prognostic state of various kidney diseases in human and animal models such as acute kidney injury (AKI), chronic kidney disease (CKD), diabetic nephropathy (DN), polycystic kidney disease, and renal cell carcinoma (Portela and Esteller, 2010; Takawa et al., 2011; Dressler and Patel, 2015; McGrath and Trojer, 2015; Cai et al., 2016; Siddiqi et al., 2016). This review focuses on recent reports on the expression and role of histone methyltransferases in various kidney diseases and their potential as targets for the treatment of these diseases.

### EZH2

EZH2, the functional enzymatic component of polycomb repressor complex 2 (PRC2), is responsible for the trimethylation of histone H3 lysine 27 (H3K27me3). EZH2 and the whole PRC2 are critical for regulating a number of genes involved in development and tumorigenesis. In the kidney, EZH2 transcripts are detectable throughout embryonic development (Caretti et al., 2004), but absent in adult kidneys. During renal development, a moderate level of EZH2 protein is detectable in the nuclei of renal tubular epithelial cells at E14.5, but not in glomeruli; in fetal kidneys at E18.5, a small amount of EZH2 protein is also observed in the cytoplasm of renal tubular epithelial cells (Ningxia et al., 2011). The localization of EZH2 protein within cells at different stage of development suggests its distinct regulatory role at early and later periods of organogenesis. EZH2 has been reported to be required for both cellular proliferation and differentiation in the process of organogenesis (Martinez and Cavalli, 2006; Oktaba et al., 2008).

Increasing evidence indicates that the abnormal expression or activity of EZH2 is associated with the development of chronic renal disease. In a murine model of renal fibrosis induced by unilateral ureteral obstruction, EZH2 is expressed in myofibroblasts and injured renal tubular cells. Similarly, increased EZH2 levels have been detected in those two cell types in kidney biopsy samples of patients with focal segmental glomerulosclerosis and IgA nephropathy, but not with minimal change disease (Zhou et al., 2016) (**Table 3**). Mechanistic studies showed that downregulation of EZH2 by inhibitors or siRNA reduces the activation of renal interstitial fibroblasts and epithelial-to-mesenchymal transition (*EMT*) of renal tubular cells *in vitro*; treatment with the EZH2 inhibitor 3-deazaneplanocin A (3-DZNeP) also attenuates UUO-induced renal fibroblast activation, partial EMT and fibrosis in mice (Zhou et al., 2016; Zhou et al., 2018a). The antifibrotic actions of EZH2 blockade are associated with dephosphorylation of multiple profibrotic receptors, including epidermal growth factor receptor and platelet derived growth factor receptor and inactivation of several intracellular signaling pathways, including Smad3, signal transducer and activator of transcription 3 (STAT3), extracellular signal-regulated kinase ½ (ERK1/2) and AKT (Vignais et al., 1996; Wang et al., 2005; Zhou et al., 2016). Moreover, we found that EZH2 expression is required for renal epithelial cells arrested at the G2/M phase of the cell cycle (Zhou et al., 2016; Zhou et al., 2018a) to change into a cell phenotype that is able to produce and release an excessive amount of growth factors and cytokines, thereby inducing the transition of fibroblasts into myofibroblasts (Grande et al., 2015; Lovisa et al., 2015). As development of partial EMT in renal epithelial cells is driven by Snail1 and Twist, two transcriptional factors, we also examined the role of EZH2 in the regulation of their expression following UUO injury. We showed that blocking EZH2 with 3-DZNeP led to decreased expression of Snail1 and Twist (Zhou et al., 2018a). Collectively, these data suggest that EZH2 is critically involved in the regulation of renal fibroblast activation, partial EMT and renal fibrosis. In contrast to our observations, it appears that EZH2 protects against podocyte oxidative stress and renal injury in diabetes. This is evidenced by data showing that pharmacologic or genetic depletion of EZH2 induced podocyte injury and augmented oxidative stress and proteinuria in diabetic mice (Siddiqi et al., 2016) (**Table 2**). Furthermore, genetic depletion and pharmacological inhibition of EZH2 promotes podocyte injury and glomerular in murine models of nephrotoxicity and subtotal nephrectomy (Majumder et al., 2018) (**Table 2**). The distinct functions of EZH2 in renal tubules and podocytes are not clear, but may reflect differences in location and targets in the kidney. In podocytes, EZH2 upregulation leads to repression of endogenous antioxidant inhibitor thioredoxin interacting protein (TxnIP), a molecule that inhibits the actions of the endogenous antioxidant thioredoxin and promotes oxidative injury (Siddiqi et al., 2016).

**Table 2:** Histone methyltransferase inhibition in kidney diseases.

KMTs	Histone targets	Inhibitors or Knockout mice	Model	Effects	References
EZH2	H3K27	3-DZNeP	UUO	Inhibits renal fibroblast activation and EMT, alleviates renal fibrosis	(Zhou et al., 2016; Zhou et al., 2018a)
		3-DZNeP	I/R or FA induced AKI	Alleviates AKI and reduces renal tubular cell death	(Zhou et al., 2018b)
		3-DZNeP	Cisplatin induced AKI	Protects against renal tubular cell death, inhibits AKI	(Ni et al., 2019)
		3-DZNeP	Hyperuricemia-induced CKD	Inhibits fibroblast activation and proliferation, attenuates renal injury	(Shi et al., 2019)
		3-DZNeP KO mice EPZ-6438, KO mice	STZ-induced DN, Adriamycin nephrotoxicity, SNx	Induces podocyte injury, increases oxidative stress and proteinuira Sensitize mice to glomerular disease increase podocyte injury and dedifferentiation	(Siddiqi et al., 2016)
			Same models as above	Sensitize mice to glomerular disease increase podocyte injury and dedifferentiation	(Majumder et al., 2018)
		GSK126	Rat renal interstitial fibroblast cells	Inhibits activation of cultured renal interstitial fibroblasts	(Zhou et al., 2016)
G9a	H3K9	BIX01294	UUO	Attenuates renal fibrosis and retains klotho expression	(Irifuku et al., 2016)
SUV39H1	H3K9	Chaetocin	Mesangial cells	Increases fibronectin and p21expression	(Lin et al., 2016)
SET7/9	H3K4	Sinefungin	UUO	Ameliorates renal fibrosis; Suppresses peritoneal fibrosis	(Sasaki et al., 2016)
DOT1L	H3K79	EPZ5676	UUO	Inhibits renal fibroblast activation and EMT; attenuates renal fibrosis	(Liu et al., 2019)
SMYD2	H3K4;H3K36	AZ505 KO mice	Hypomorphic Pkd1^nl^/^nl^ mice	Delay cyst growth in ADPKD, reduce activation of STAT3 and NF-κB	(Li et al., 2017)

DN, Diabetic nephropathy; STZ, streptozotocin; CKD, chronic kidney disease; UUO, unilateral ureteral obstruction; SNx, subtotal nephrectomy; KO, knockout mice; AKI, acute kidney injury; EMT; epithelial–mesenchymal transition; ADPKD, autosomal dominant polycystic kidney disease; STAT3, signal transducer and activator of transcription 3; NF-κB, nuclear factor kappa light chain enhancer of activated B cells.

EZH2 also contributes to the pathogenesis of acute kidney injury (AKI). In mouse models of ischemia/reperfusion or folic acid-induced AKI, EZH2 and H3K27 are highly expressed in the injured tubules. Pharmacological inhibition of EZH2 lessens pathological changes and attenuates renal tubule injury and cell death; inhibition of EZH2 with either 3-DZNep or its specific siRNA also promotes survival of renal epithelial cells in culture following oxidant injury (Zhou et al., 2018b). Moreover, EZH2 inhibition increases expression of its multiple target molecules, including E-cadherin, TIMP-2/3, and RKIP, which are associated with renal tubular injury (Zhou et al., 2018b). In line with our observations, Liang et al. (2019) demonstrated that treatment with 3-DZNep improves renal dysfunction, lessens tubular injury, reduces production of several cytokines, including TNF-α, MCP-1 IL-6 and IL-18 and decreases activation of p38, a stress protein in mice after ischemia/reperfusion. Furthermore, Li et al. (2016a) reported that inhibition of EZH2 with 3-DZNep resulted in amelioration of early acute renal allograft rejection in rats through suppression of activation of alloreactive T cells and subsequent production of multiple inflammatory cytokines. Thus, EZH2 is critically involved in the acute inflammatory response and renal injury. Recently, we further demonstrated that administration of 3-DZNep was protective in a murine model of AKI induced by cisplatin through a mechanism involved in the preservation of E-cadherin (Ni et al., 2019).

Collectively, these studies indicate that EZH2 plays a detrimental role in renal tubules but a protective role in podocytes. Therefore, targeting EZH2 may be a potential approach for the treatment of renal fibrosis, AKI and acute rejection after transplantation, but not for diabetic nephropathy. Given that EZH2 plays a protective role in podocytes, EZH2 inhibitors should be avoided in renal diseases characterized by podocyte injury.

### G9a

Among the histone modification enzymes, G9a is a key histone lysine methyltransferase that can catalyze mono- and dimethylation at H3K9 (me1 and me2), a repressive chromatin marker (Tachibana et al., 2001; Tachibana et al., 2002). In the developing kidney, G9a is mainly expressed in the Pax2^+^ cap mesenchyme and the distal and proximal portions of nascent nephrons (McLaughlin et al., 2014). Several studies have revealed that G9a is upregulated in various human tumors, including colorectal cancer, breast cancer, ovarian cancer, and bladder cancer (Huang et al., 2010; Hua et al., 2014; Zhang et al., 2015; Chen et al., 2017; Cao et al., 2019) and plays a role in tumorigenesis. G9a activity can also be induced by transforming growth factor-β1 (TGF-β1) and is involved in the development of epithelial mesenchymal transposition in cancer cells (Dong et al., 2012). Given that TGF-β1 is a pivotal cytokine in tissue fibrosis, Irifuku et al. (2016) examined the expression of G9a and its role in a murine model of UUO-induced renal fibrosis by administration of G9a small interfering RNA and BIX01294, a specific inhibitor of G9a. They found that G9a expression levels were upregulated in the murine kidney of UUO and in human kidney biopsy specimens obtained from patients with IgA nephropathy (**Table 3**). Treatments with interfering RNA and BIX01294 reduced activation of renal interstitial fibroblasts and accumulation of ECM proteins, which is accompanied by increased klotho expression and decreased methylation of H3K9. *In vitro*, they also observed that treatment with BIX01294 inhibited TGF-β1-induced fibrotic changes and klotho downregulation in renal epithelial cells (Irifuku et al., 2016). In a separate study, the same group found that pharmacological inhibition of G9a attenuated peritoneal fibrosis and improved peritoneal function in a murine model of peritoneal fibrosis induced by methylglyoxal (Maeda et al., 2017). Nevertheless, the regulatory role of G9a is not limited to renal and peritoneal fibrosis. G9a has also been reported to play a role in the pathogenesis of idiopathic pulmonary fibrosis (Coward et al., 2018). Thus, G9a may mediate tissue fibrosis in different organs, but there are so far no reports about its role in acute organ injury. One group, however, reported that BIX01294 treatment protects hair cells from aminoglycoside-induced damage in mice (Yu et al., 2013), suggesting that like EZH2, G9a may also be involved in the pathogenesis of AKI. Further investigation into this issue is needed.

**Table 3:** Expression of HMTs in human kidney diseases.

Human kidney disease	HMTs		Histone Targets	References
Focal segmental glomerulosclerosis, IgA nephropathy	EZH2 EZH2	upregulated upregulated	H3K27 H3K27 me3	(Zhou et al., 2016; Zhou et al., 2018a)
IgA nephropathy	G9a	upregulated	H3K9	(Irifuku et al., 2016)
Diabetic nephropathy	SUV39H1	upregulated	H3K7	(Wang et al., 2018b)
IgA and membranous nephropathy	SET7/9	upregulated	H3K4	(Sasaki et al., 2016)
Polycystic kidneys	SMYD2	upregulated	H3K4, H3K36	(Pires-Luis et al., 2015; Li et al., 2017)
Membranous nephropathy Focal segmental glomerulosclerosis	MLL3 EZH2	Upregulated upregulated	H3K4 H3K36 H3K27	(Fujino and Hasebe, 2016) (Wang et al., 2019)

### SUV39H1

The histone methyltransferase SUV39H1 catalyzes the trimethylation of histone H3 lysine 9 (H3K9me3) and is a well-known transcriptional repressor of inflammatory genes. The impact of SUV39H1 on inflammatory gene promoters under the stimulation of high-glucose has been reported in various cell lines, including vascular smooth muscle cells (VSMCs) (Villeneuve et al., 2008), macrophages (Li et al., 2016b), and cardiomyocytes (Yang et al., 2017b). Recent studies suggest that SUV39H1 and H3K9me3 were overexpressed in renal tubules of patients with diabetic nephropathy (Wang et al., 2018b) (**Table 3**), indicating that their expression correlates with the development of chronic kidney disease. However, hyperglycemia decreased expression of SUV39H1 in type 1 diabetes-associated renal fibrosis (Goru et al., 2016). Further studies showed that high glucose-induced downregulation of H3K9me3 and SUV39H1 primarily occurred in diabetic mouse vascular smooth muscle cells and mesangial cells (Lin et al., 2016). Interestingly, genetic or pharmacological inhibition of phosphoinositide 3-kinase (PI3K) reduced high glucose-induced downregulation of SUV39H1 in mesangial cells, while genetic overexpression of SUV39H1 or pharmacological increase of SUV39H1 was protective against the progression of DN (Lin et al., 2016). These findings imply that dysregulation of SUV39H1 may contribute to DN progression and that SUV39H1 may serves as a therapeutic target for the treatment of DN.

### Dot1/DOT1L

The lysine methyltransferase responsible for H3K79 methylation is named by Dot1 (disruptor of telomeric silencing 1) in lower eukaryotes and Dot1-like protein (DOT1L) in mammals (Min et al., 2003). It is the only known mammalian histone methyltransferase (Min et al., 2003) that can catalyze the mono-, di-, and tri- methylation of histone H3 lysine 79 (H3K79) and regulate the activation or repression of gene transcription (Min et al., 2003). The mono- and di-methylation of H3K79 is associated with gene transcriptional activation, whereas H3K79 trimethylation is associated with gene repression (Wong et al., 2015). As one of the most studied histone methyltransferase, DOT1L has been shown to play a vital role in many biological processes, including mammalian development, cell cycle progression, cardiomyocyte differentiation, postembryonic development, somatic reprogramming, DNA damage response, and tumorigenesis (Huyen et al., 2004; Janzen et al., 2006; Jones et al., 2008; Wakeman et al., 2012; Wen et al., 2015; Cattaneo et al., 2016). It has been reported that increasing DOT1L expression by spironolactone or by stable transfection led to impaired endothelin-1 expression in NRK-52 cells, indicating a possible role of DOT1L in mediating the pathogenesis of diabetes (Wu et al., 2016). Further studies showed that spironolactone attenuates kidney injury by restoring DOt1a, one of five splicing variants (Dotla-Dotle) of DOT1L, which mediates repression of endothelin-1 expression in streptozotocin-induced diabetic rats (Zhou et al., 2012). In addition, Dotla plays a role in the regulation of water homeostasis by targeting aquaporin 5 (Wu et al., 2013). Although the role of DOT1L in most kidney diseases is not known currently, our recent studies demonstrated that DOT1L was critically involved in the pathogenesis of renal fibrosis in a murine model of UUO-induced chronic kidney disease. DOT1L and di-methylated H3K79 were highly expressed in renal tubular epithelial cells and myofibroblasts in UUO kidneys and in *in vitro* cultures of these two types of cells; pharmacological inhibition of DOT1L with EPZ5676, a highly selective inhibitor of DOT1L, significantly reduced renal fibrosis (Liu et al., 2019). Treatment with EPZ5676 or DOT1L small interfering RNA also inhibited TGF-β1 and serum-induced activation of renal interstitial fibroblasts and epithelial-mesenchymal transition (EMT) *in vitro*. Currently, DOT1L expression in human kidney disease remains unclear and requires further investigations.

### SET7/9

SET7/9 is a protein lysine methyltransferase that methylates histone H3K4 and some nonhistone proteins such as p53 (Kurash et al., 2008). Previous studies revealed a close association between SET7/9 and neoplasms such as breast cancer, hepatocellular carcinoma, prostate cancer, and colorectal cancer (Chen et al., 2016; Huang et al., 2017; Duan et al., 2018; Wang et al., 2018a). Recently, several studies indicated that SET7/9–mediated H3K4 methylation is also related to renal disease. TGF-β1-induced up-regulation of ECM-associated genes was accompanied by increased expression of H3K4me1/2/3 and SET7/9 (Sun et al., 2010); the level of H3K4me1 and SET7/9 recruited at MCP-1 promoters were increased in the kidneys of db/db mice (Chen et al., 2014a); SET9 regulates TGF-β1-induced activation of renal fibroblasts through promoting Smad-3 activation (Shuttleworth et al., 2018). SET7/9 knockdown by small interfering RNAs (siRNAs) significantly inhibited MCP-1 expression and improved glomerular lesions, indicating that SET7/9-mediated H3K4me1 is involved in the expression of MCP-1 in the kidneys of db/db mice (Chen et al., 2014a). Another study showed that TGF-β1 induced the recruitment of SET7/9 to the p21 gene promoter, while SET7/9 gene silencing with siRNAs significantly attenuated TGF-β1-induced p21 gene expression in rat mesangial cells (Guo et al., 2016). Further studies showed that inhibition of SET7/9 with sinefungin, a selective inhibitor of SET7/9 or siRNA attenuated renal fibrosis, as indicated by decreased expression of mesenchymal markers and ECM proteins in kidneys of UUO mice; sinefungin can also inhibit expression of TGF-β1-induced α-smooth muscle actin expression in cultured renal epithelial cells and renal interstitial fibroblasts (Sasaki et al., 2016). Interestingly, expression of SET7/9 was also positively correlated with the degree of interstitial fibrosis in human kidneys of patients with IgA and membranous nephropathy (Sasaki et al., 2016) (**Table 3**). These studies provide evidence for the involvement of SET7/9 in renal fibrosis. Sasaki et al. (2016) reported that SET7/9 expression was also increased in the peritoneum in a murine model of peritoneal fibrosis induced by methylglyoxal and as well as in non-adherent cells isolated from the effluent of PD patients; treatment with sinefungin inhibited expression of collagen deposition in the peritoneum and improved peritoneal function, which is accompanied by decreased expression of H3K4me1 as well (Tamura et al., 2018).

Moreover, SET7/9 plays a role in inflammation and diabetes. Increased inflammatory gene expression and SET7/9 recruitment in macrophages were observed in diabetic mice; targeted silencing of SET7/9 with siRNA reduced TNF-α-induced recruitment of NF-κB p65 to inflammatory gene promoters (Li et al., 2008). In addition, hyperglycemia increased expression of SET7/9 in rat mesangial cells and in the kidney of type 1 diabetic rats (Goru et al., 2016). These findings suggest the possible role of SET7/9 in the development of renal fibrosis in diabetic kidney disease, but further investigation is needed to understand the mechanism of action of SET7/9.

Taken together, these results demonstrate that upregulation of SET7/9 and its related H3K4me1 is associated with the pathogenesis of renal and peritoneal fibrosis. Targeting SET7/9 may be a novel therapeutic treatment for fibrotic diseases.

### SMYD2

SMYD2, one of the SET and MYND-containing lysine methyltransferases (SMYD), can methylate both H3K4 and H3K36 and non-histone proteins, including p53/TP53 and RB1 (Tracy et al., 2018). Increased expression of SMYD2 was initially identified in renal tumor tissue (Pires-Luis et al., 2015). Recently, Li et al. (2017) made an interesting observation that SMYD2 was also highly expressed in the polycystic renal tubular epithelial cells of autosomal dominant polycystic kidneys in a mouse model and human tissue. By utilizing Pkd1-knockout mice and AZ505, a specific inhibitor of SMYD2, they demonstrated that SMYD2 is a critical mediator of renal cyst growth. Moreover, SMYD2 induced cystic renal epithelial cell proliferation while survival was found to be mediated by STAT3 and the p65 subunit of NF-κB; activation of these two pathways is also required for the expression and activation of SMYD2 (Li et al, 2017) (**Table 3**). These data suggest two positive feedback loops, SMYD2/STAT3/SMYD2 and SMYD2/NF-κB/SMYD2 in cyst development. Currently, the role of SMYD2 in other kidney diseases remains to be reported. While it has been shown that SMYD2 is also highly expressed in human kidney tumors (Pires-Luis et al., 2015), there is no report about SMYD2 expression in other human kidney diseases so far.

### Other Histone Methyltransferases Associated With Kidney Diseases

Other methyltransferases have also been reported to be involved in the development of kidney diseases and pathological conditions. Wang et al. (2019) showed that overexpression of EZH1 inhibited injury of cultured renal tubular cells (HK2) in response to aristolochic acid. Fujino and Hasebe (2016) demonstrated that histone H3K4 me3 levels were elevated in the podocytes of kidneys in patients with membranous nephropathy (**Table 3**) and mice treated with *lipopolysaccharide* (LPS); treatment with siRNA specific to MLL3 (an H3K4 methyltransferase) ameliorated podocyte swelling, reduced proteinuria and improved renal function in mice treated with LPS. This suggests that MLL3 expression and H3K4 methylation may be involved in LPS-induced podocyte injury and proteinuria. Whether MLL3 also plays a role in the pathogenesis of human membranous nephropathy needs further investigation.

Recently, sequencing of whole exomes from patients with sporadic or familial focal and segmental glomerulosclerosis (FSGS) identified three new genes (SCAF1, SETD2, and LY9) that are located closer to known FSGS genes. Among them, SETD2 is a histone H3 lysine 36 methyltransferase, suggesting that SETD2 may contribute to FSGS. Additional studies are required for elucidating the role of SETD2 in this disease.

### Representative Histone Methyltransferase Inhibitors

Given the fact that many HMTs are implicated in the pathogenesis of kidney diseases, targeting HMTs by small-molecule modulators could be effective therapy for treating them. In the past decade, many small molecules that target histone methyltransferases have been developed and used for treatment of tumors in animal models, and some of them have advanced to clinical trials. Similarly, several the histone methyltransferases inhibitors have been tested for their efficacy in the treatment of experimental kidney diseases (**Table 2**). Here, we summarize and highlight those histone methyltransferase inhibitors widely used in animal models of kidney diseases.

### EZH2 Inhibitors

Among the EZH2 inhibitors, 3-DZNeP is frequently used in animal studies of various diseases. It can induce degradation of EZH2 and subsequently inhibits its activity. Our studies show that treatment with 3-DZNeP dose-dependently inhibited activation and proliferation of renal interstitial fibroblasts *in vitro*. Administration of 3-DZNeP at as low as 2 mg/kg can effectively attenuate renal fibrosis, inhibit inflammation and lessen acute kidney injury with improvement of renal function. However, DZNeP is not a direct EZH2 inhibitor and exerts its inhibitory action through blocking S-adenosyl-L-homocysteine hydrolase. Thus, its application may lead to unwanted outcomes in SAM-dependent reactions. Moreover, the lack of specificity and short half-life also limit its translation to clinical applications.

Recently, Some potent and more selective EZH2 inhibitors have been developed. Those inhibitors include GSK926, GSK-343, EPZ-6438, EPZ-005687, EPZ-011989, EI1, UNC-1999, and CPI-169. All are competitive inhibitors of EZH2. Among them, GSK126 is the most potent inhibitor of EZH2 . That can effectively inhibited activation of cultured renal interstitial fibroblasts (Zhou et al., 2016). In addition to the competitive inhibition of EZH2, a novel strategy to suppress EZH2 by protein degradation has been developed. For example, GNA022 can covalently bind to cYS 668 at the set domain of EZH2 and induce EZH2 degradation, thereby inhibiting tumor growth (Wang et al., 2017).

It is important to note that EPZ-6438, GSK126, and CPI-1205 have already entered clinical trials (Trial number: NCT01897571, NCT02601950, NCT02601937, NCT02395601, and NCT02082977). It was found that they are well tolerated and have antitumor activity in patients with refractory B-cell non-Hodgkin lymphoma and advanced solid tumors (Yan et al., 2016). Despite great progress in clinical trials in determining the potential of EZH2 inhibitors as cancer therapy, the efficacy of those inhibitors in animal models of kidney diseases and in human kidney diseases hasn’t yet been shown.

### G9a Inhibitors

A number of small molecule inhibitors have been developed with the capacity to inhibit G9a catalytic activity and have been used in various *in vitro* and *in vivo* experiments. BIX01294 (diazepin-quinazolin-amine derivative), one of the first molecules developed to target G9a, is a competitive inhibitor specific for G9a that can reduce G9a-mediated H3K9 di-methylation, but not mono-methylation (Kubicek et al., 2007). Unlike many other HMT inhibitors, BIX-01294 competes with G9a substrate and not with G9a cofactor *S*-adenosyl-methionine (SAM), the source of the transferred methyl group. Since BIX-01294 has an intrinsic toxic effect, researchers have developed a second G9a inhibitor, UNC0638, which has high potency and specificity for G9a with lower cell toxicity. In addition, UNC0638 exhibited higher lipophilic characteristics and cell membrane permeability. Both BIX01294 and UNC0638 have been reported to inhibit cellular proliferation in various cancer cell lines, such as breast, squamous head and neck carcinoma (Artal-Martinez de Narvajas et al., 2013; Savickiene et al., 2014; Ueda et al., 2014). However, pharmacokinetics of UNC0638 is poor. Recently, Liu et al. (2013) have developed a new compound, UNC0642 that is suitable for animal studies. UNC0642 has better pharmacokinetics and maintains a high selectivity and low cell toxicity. As described in the previous section, inhibition of G9a with BIX01294 has been reported to attenuate renal fibrosis and peritoneal fibrosis in animal models. BIX01294-mediated inhibition of renal fibrosis is associated with increased expression of klotho, a renoprotective inhibitor (Irifuku et al., 2016), but BIX01294 treatment did not alter expression of klotho in renal epithelial cells (Maeda et al., 2017). The efficacy of UNC0638 and UNC0642 has not yet been evaluated in renal diseases.

### SUV39H1 Inhibitors

Like methyltransferases G9a, SUV39H1 is also a H3K9 enzyme. G9a catalyzes H3K9 me0 to me1 and me1 to me2 (Tachibana et al., 2002) while SUV39H1 catalyzes di- to tri-methylation of H3K9 (Lachner et al., 2001). Tri-methylated H3K9 generates a binding site for the HP1 protein, which leads to inhibition of gene transcription. Currently, chaetocin, a fungal mycotoxin originally isolated from *Chaetomium minutum*, has been reported to be a specific inhibitor of SUV39H1 (Greiner et al., 2005). However, subsequent studies showed that at higher concentration of chaetocin also inhibits G9a activity, suggesting it may be a nonspecific inhibitor of HMTs (Cherblanc et al., 2013). This compound has been used in both *in vitro* and *in vivo* studies It exhibits a potent cytotoxic effect in acute myeloid leukemia cells (Lai et al., 2015) and a protective effect on the heart in a murine model of cardiac injury following myocardial infarction (Yang et al., 2017a) and brain injury following cerebral ischemia (Schweizer et al., 2015). In contrast to those findings, overexpression of SUV39H1 in the kidney of db/db models reversed the diabetic phenotype (Villeneuve et al., 2008), and administration of chaetocin increased fibronectin and p21(WAF1) protein levels in cultured mouse mesangial cells exposed to high glucose at the concentration that reduced histone H3K9me3 levels (Lin et al., 2016). As such, it appears that SUV39H1 inhibition potentiates renal injury, at least in the mouse model of DN. To date, there is still no data on the possible involvement of SUV39H1 in other animal models of kidney disease.

### SET7/9 Inhibitors

Sinefungin is a small molecule inhibitor of SET7/9 that acts by competing with S-adenosyl-L-methionine and can ameliorate renal fibrosis (Sasaki et al., 2016) and peritoneal fibrosis (Tamura et al., 2018) in animal models. However, sinefungin treatment significantly reduced expression of H3K4me1, but did not alter expression of H3K4me2 and H3K4me3 in the kidneys of UUO mice. In addition to sinefungin, cyproheptadine, a clinically approved antiallergy drug, has recently been identified as a Set7/9 inhibitor using a fluorogenic substrate-based HMT assay. This compound can bind Set7/9 and inhibit its enzymatic activity (Takemoto, et al., 2016). The efficacy of cyproheptadine in inhibiting tissue fibrosis, including renal fibrosis remains to be determined.

### DOT1l Inhibitors

EPZ004777, EPZ5676, and SGC0946 have been reported to inhibit DOT1L. All act as competitive inhibitors of SAM, the cofactor required for the methyltransferase activity of DOT1L. EPZ004777 was first developed by Epizyme Inc. as an inhibitor of DOT1L. It shows a remarkable selectivity against other histone methyltransferases and selectively kills MLL-rearranged leukemia cells in culture (Daigle et al., 2011). Its poor pharmacokinetic properties, however, made this compound unsuitable for animal study and clinical development. A second generation DOT1L inhibitor, EPZ5676, has improved pharmacokinetic properties (Daigle et al., 2013) and has been used in animal studies and clinical trials. A first phase I study of EPZ5676 with relapsed/refractory acute leukemia has been completed; drug administration is well tolerated, and only about 15% of treated patients display adverse events. As indicated in the previous section, we also found that EPZ5676 protects against renal fibrosis. But EPZ-5676 shows low oral bioavailability and needs continuous infusion or subcutaneous injection. Both infusion and subcutaneous injection are well tolerated in animals with no overt signs of toxicity (Daigle et al., 2013). Metabolic studies showed that EPZ5676 is primarily excreted in feces and has low renal excretion (Waters et al., 2016), making EPZ5676 a suitable agent for being developed as a drug to treat kidney disease.

### SMYD2 Inhibitors

LLy-507 has been reported as a potent cell-permeable and selective small molecule inhibitor of SMYD2. It has 100-fold selectivity for SMYD2 over 24 other protein or DNA methyltransferases (Nguyen et al., 2015). Reports have shown that LLy-507 was effective in inhibiting proliferation of cancer cell lines in esophageal, liver, and breast cancers (Tracy et al., 2018). In the kidney, AZ505 has been reported to be effective in inhibiting cyst growth in a murine model of autosomal dominant polycystic kidney disease (Li et al., 2017).

### Conclusion and Future Perspectives

Epigenetic regulation plays a vital role in the modulation of gene expression under normal and pathological conditions. Emerging evidence has shown increased expression of some HMTs such as EZH2, G9a, SUV39H1, and SET7/9 in several kidney diseases. Pharmacological targeting of those histone methyltransferases has been shown to attenuate renal fibrosis in animal models. Some of these molecules are also effective in interfering with the progression of diabetic nephropathy. This indicates that targeting histone methylation could be a promising therapeutic strategy for the treatment of kidney diseases. However, multiple histone methyltransferases are activated and involved in a given pathological process, and a crosstalk exists between different methylated sites, strongly suggesting that targeting one histone methyl residue or a related enzyme may lead to unpredictable effects. For example, a majority of KMTs contain a SET domain, and most inhibitors have been developed to target this domain, pointing to a potential lack of specificity leading to unwanted effects. Therefore, it is necessary to conduct further mechanistic investigations to complete the map of histone methylation in a given renal disease and develop inhibitors with higher specify and satisfactory pharmacokinetics. Moreover, since current pre-clinical studies related to HMT inhibitor are only conducted in limited animal models of renal disease, more research is needed to evaluate the efficacy and side effects of HMT inhibitors in different models of renal diseases and compare their efficacy and effects with those in other organs. Finally, although clinical trials of some EZH2 and DOT1L inhibitors for treatment of tumors have been reported (Mohammad et al., 2019), there are still no clinical trials for HMT inhibitors in renal diseases. With more evidence showing the efficacy of HMT inhibitors in animal models of kidney diseases, clinical trials with selected HMT inhibitors in renal disease are worthy of exploration.

## Author Contributions

CY drafted the manuscript and SZ edited it. Both authors approved it for publication.

## Conflict of Interest

The authors declare that the research was conducted in the absence of any commercial or financial relationships that could be construed as a potential conflict of interest.
